# 重组人血管内皮抑素对非小细胞肺癌淋巴管生成的抑制及对循环肿瘤细胞的影响

**DOI:** 10.3779/j.issn.1009-3419.2014.10.03

**Published:** 2014-10-20

**Authors:** 立群 尚, 皆 赵, 伟 王, 旺 肖, 军 李, 学昌 李, 伟安 宋, 军强 刘, 锋 文, 彩迎 岳

**Affiliations:** 100048 北京，海军总医院胸外科 Department of Thoracic Surgery, PLA Navy General Hospital, Beijing 100048, China

**Keywords:** 重组人血管内皮抑素, 荷瘤模型, 微淋巴管密度, 表达阳性率, 循环肿瘤细胞, Recombinant human endostatin injection, Tumor-bearing model, Microlymphatic vessel density, Positive expression rate, Circulating tumor cells

## Abstract

**背景与目的:**

血管内皮抑素可以抑制肿瘤新生血管的生成，但对肿瘤微淋巴管的形成与发展是否存在抑制效应引起我们关注。本研究旨在探讨重组人血管内皮抑素（recombinant human endostatin injection）对非小细胞肺癌组织中血管内皮生长因子（vascular endothelial growth factor, VEGF）-C、VEGF-D和VEGF受体（VEGFR）-3表达及对外周血循环肿瘤细胞数目的影响。

**方法:**

荷瘤裸鼠随机分为空白对照组、顺铂组、不同浓度重组人血管内皮抑素组及重组人血管内皮抑素+顺铂组，连续给药2周。1周后检测肿瘤组织中VEGF-C、VEGF-D、VEGFR-3的表达水平和微淋巴管密度。免疫荧光染色诊断和计数循环肿瘤细胞。

**结果:**

重组人血管内皮抑素组与重组人血管内皮抑素联合顺铂组的表达阳性率、微淋巴管密度均明显低于空白对照组与顺铂组（*P* < 0.05）；较高浓度的重组人血管内皮抑素联合顺铂组与重组人血管内皮抑素组表达阳性率和微淋巴管密度低于相应较低重组人血管内皮抑素浓度的组别（*P* < 0.05）。各组微淋巴管密度与VEGF-C、VEGF-D、VEGFR-3表达阳性率存在正相关。重组人血管内皮抑素联合顺铂各组的循环肿瘤细胞数目明显低于单独使用顺铂或重组人血管内皮抑素（*P* < 0.05）。

**结论:**

重组人血管内皮抑素可以抑制肿瘤新生淋巴管生成，减少循环肿瘤细胞，作用大小与浓度有关。与顺铂联合使用能够更有效的减少循环肿瘤细胞。

## Abstract (en)

**Background and objective:**

It is unclear how could endostatin effect tumor lymphangiogenesis? The aim of this study is to explore inhibitory effect of recombinant human endostatin injection (endostar) on lymphangiogenesis in non-small cell lung cancer (NSCLC) tissue and its effect on circulating tumor cells (CTC) in peripheral blood.

**Methods:**

Tumor-bearing model nude mice were divided into eight groups randomly (*n*=7), including control group, cisplatin group, several concentration endostar groups and endostar plus cisplatin groups. Continuous administration of Endostar for two weeks, observed one week after the end of administration. Using HE staining and immunohistochemical staining to diagnose the tumor tissue and suspect metastasis lymph nodes, detected vascular endothelial growth factor (VEGF)-C, VEGF-D, VEGF receptor (VEGFR)-3 expression level and microlymphatic vessel density (MLVD) of tumor tissue. Enrichment of circulating tumor cells in peripheral blood used immunomagnetic negative selection strategy, used immunofluorescence staining to diagnose and count CTCs.

**Results:**

Microlymphatic vessel density and the positive expression rate of VEGF-C, VEGF-D, VEGFR-3 in three endostar groups and three endostar plus cisplatin groups were significantly less than those in control group and cisplatin group. Microlymphatic vessel density and the positive expression rate of VEGF-C, VEGF-D, VEGFR-3 in endostar plus cisplatin group and endostar group with high endostar concentration were significantly less than those with low endostar concentration; There was a significant positive correlation between microlymphatic vessel density and the positive expression rate of VEGF-C, VEGF-D, VEGFR-3. The number of circulating tumor cells in endostar plus cisplatin groups were significantly less than that of endostar or cisplatin alone.

**Conclusion:**

Endostar could inhibit tumor lymphangiogenesis and reduce tumor cells into the bloodstream through the lymphatic. Inhibitory effect concerned with drug concentrationwith a dose-dependant.

非小细胞肺癌（non-small cell lung cancer, NSCLC）约占肺癌发病率的80%，多通过淋巴、血液及肿瘤浸润等方式进行局部侵犯和远处转移。研究^[1]^表明，淋巴转移在肿瘤的早期转移中比血行转移更为重要，如何阻断肿瘤淋巴转移是控制肺癌进展的重要课题。肺癌循环肿瘤细胞（circulating tumor cell, CTC）的研究^[2-7]^发现检测肺癌CTC有助于早期诊断及判断患者预后，与临床分期有关，在治疗期间定期检测并监测CTC的变化，可为评价治疗方案的敏感性提供依据，有利于开展个体化的治疗。通过CTC检测来早期发现和诊断肿瘤已成为肿瘤研究的一个趋势^[8]^。重组人血管内皮抑素是由我国自主研发的抗癌新药，为内源性血管生成抑制剂，能够选择性抑制血管内皮细胞的增殖与迁移，达到抑制肿瘤形成新生血管、切断肿瘤细胞的营养供给途径、诱导肿瘤细胞凋亡和抑制肿瘤增殖或转移的目的^[9, 10]^。重组人血管内皮抑素对肿瘤血管生成的抑制作用已有较多研究与报道，但其对肿瘤淋巴管的生成是否存在类似的抑制作用，相关的临床及基础实验报道仍很少见。为此，我们设计并进行了相关研究，以期探讨重组人血管内皮抑素对NSCLC淋巴管生成及淋巴转移的作用。

## 1 材料与方法

### 1.1 材料

#### 1.1.1 A549肺癌细胞株

购于北京协和基础实验室细胞库，细胞培养在海军总医院中心实验室，采用含10%胎牛血清的DMEM/F12（1:1）培养基培养，细胞培养环境为5% CO_2_，37.5 ℃恒温培养箱。

#### 1.1.2 BALB/C裸鼠

5周龄，雌雄各半，体重均为22 g-25 g，购于军事医学科学院实验动物中心，裸鼠饲养在解放军304医院SPF级动物实验室，在环境控制条件下饲养，温度（22±1）℃，12 h光亮/黑暗环境，6:00-18:00为光亮周期，18:00-6:00为黑暗周期，并于实验室自由饮食。动物实验获得海军总医院伦理委员会批准。

#### 1.1.3 药物与试剂

重组人血管内皮抑素，由山东先声麦得津生物制药有限公司提供；顺铂由山东齐鲁制药有限公司提供；DMEM/F12（1:1）培养基，胎牛血清，胰蛋白酶及细胞培养耗材均购自北京圣希康生物科技有限公司；鼠抗血管内皮生长因子（vascular endothelial growth factor, VEGF）-C、VEGF-D和VEGF受体（VEGFR）-3单克隆抗体试剂盒购于美国Santa Cruz公司；鼠抗anti-podoplanin Protein/gp36试剂盒，PV-6000通用型免疫组化检测试剂盒和DAB染色试剂盒购于上海科敏生物科技有限公司；Miltenyi抗CD45免疫磁珠、LS柱、强磁场磁性细胞分离架/器购于德国美天旎公司；枸椽酸抗凝血管（ACD）、淋巴细胞分离液、细胞核荧光染料4, 6-联眯-2-苯基吲哚（DAPI）、anti-Napsin A（1:100）和anti-TTF-1（1:100）荧光抗体购于美国Sigma公司。

### 1.2 方法

#### 1.2.1 荷瘤裸鼠动物模型的建立与分组

于裸鼠右侧腋窝经皮下注射浓度为1×10^7^/mL的A549人肺癌细胞0.2 mL，建立裸鼠异种移植荷瘤模型^[11]^。注射肿瘤细胞3周后，选取56只建模成功的荷瘤裸鼠，肿瘤平均直径为（7±0.4）mm。56只成瘤裸鼠随机分为8个小组（*n*=7），各组间裸鼠平均体重，肿瘤直径无统计学差异，同组雌雄裸鼠分开饲养。空白对照组，给予尾静脉注射生理盐水0.2 mL，每天1次；顺铂组，给予尾静脉注射顺铂20 μg，隔天1次；重组人血管内皮抑素1，给予尾静脉注射重组人血管内皮抑素2 mg/kg，每天1次；重组人血管内皮抑素2，给予尾静脉注射重组人血管内皮抑素4 mg/kg，每天1次；重组人血管内皮抑素3，给予尾静脉注射重组人血管内皮抑素6 mg/kg，每天1次；重组人血管内皮抑素1+顺铂，给予尾静脉注射重组人血管内皮抑素2 mg/kg，每天1次，加顺铂20 μg，隔天1次；重组人血管内皮抑素2+顺铂，给予尾静脉注射重组人血管内皮抑素4 mg/kg，每天1次，加顺铂20 μg，隔天1次；重组人血管内皮抑素3+顺铂，给予尾静脉注射重组人血管内皮抑素6 mg/kg，加顺铂20 μg，隔天1次。所有荷瘤裸鼠均连续给药2周。

#### 1.2.2 荷瘤裸鼠的处置

给药结束后观察1周，给予水合氯醛麻醉，采用摘眼取血，每只约1 mL，为满足实验用血量每组7只动物血合并为一组共7 mL，附加实验重复上述实验使采血达到7组，所采血液置于枸橼酸抗凝管中保存；取血后处死全部裸鼠并进行完整的解剖，包括肿瘤和淋巴结解剖，以从病理学方面评估转移的存在。病理切片由海军总医院病理科制作，并对肿瘤组织和转移淋巴结进行HE染色。免疫组织化学染色诊断北京中杉金桥生物科技有限公司完成，采用免疫酶标法对肿瘤组织进行染色，检测肿瘤组织中VEGF-C、VEGF-D、VEGFR-3的表达水平和微淋巴管密度，分别采用抗VEGF-C、VEGF-D、VEGFR-3的单克隆抗体和抗鼠podoplanin蛋白抗体作为一抗，PV-6000（辣根酶标记羊抗兔/小鼠IgG多聚体）作为二抗，DAB（二氨基联苯胺）溶液作为显色剂，染色成功后组织切片中有VEGF-C、VEGF-D、VEGFR-3表达的部位和微淋巴管内皮细胞呈棕黄色。

#### 1.2.3 CTC检测

在海军总医院中心实验室完成。采用免疫磁珠负性富集法筛选出外周血循环肿瘤细胞^[12, 13]^，anti-NapsinA和anti-TTF-1荧光抗染色确定CTC ^[14]^，镜下观察CTC形态，计数各组CTC数量。CTC检测设备：激光共聚焦采用日本Olympus FV 10i；负性富集采用德国Miltenyi Midi MaCS分选器。简单的步骤如下：①免疫磁珠负性富集外周血CTCs，用缓冲液（137 mmol/L NaCl; 2.7 mmol/L KCl; 10 mmol/L Na_2_HPO_4_; 2 mmol/L EDTA; 0.5%BSA, pH 7.4）将血样转移至50 mL离心管，并定容至50 mL；450 *g*离心5 min，弃上清；②加入红细胞裂解液（155 mmol/L NH_4_Cl；10 mmol/L KHCO_3_；0.1 mmol/L EDTA）至50 mL，室温匀速孵育8 min，450 *g*离心5 min，弃上清；③重复红细胞裂解步骤，充分裂解残余的红细胞；④加入缓冲液至50 mL，细胞重悬后显微镜下计数；⑤再次450 *g*离心5 min，弃上清；⑥按10^7^个细胞加入20 μL anti-CD45磁珠的比例，将细胞与磁珠混匀，并在4 ℃避光孵育15 min；⑦加入10 mL缓冲液，450 *g*离心，弃上清；⑧按照每10^7^个细胞加入500 μL缓冲液的比例，重悬细胞；⑨细胞悬液加入已用缓冲液润洗的LS分离柱，在强磁场的作用下收集流出的细胞，滴在载玻片上，室温放置至细胞干燥；⑩加入2%多聚甲醛固定40 min，PBS洗涤3次，室温干燥后进行细胞免疫荧光染色。细胞免疫荧光染色：①细胞滴片以PBS洗涤5 min，加入0.1%的TritonX-100-PBS孵育5 min；②PBS洗涤3次，每次3 min，以2%的BSA-PBS室温封闭30 min；③加入anti-Napsin A（1:100）和anti-TTF-1（1:100）荧光抗体室温孵育1 h；④0.2%BSA-PBS洗涤3次，每次3 min；⑤细胞滴片在暗处晾干，加入7 μL的DAPI mounting medium封片，盖玻片固定，显微镜下观察并计数。染色和洗涤均在暗室中操作。

### 1.3 效果评定

组织切片免疫组织化学染色采用免疫酶标法，VEGF-C、VEGF-D、VEGFR-3蛋白免疫组织化染色阳性表达定位于肿瘤细胞及巨噬细胞的胞质，呈棕黄色颗粒，VEGFR-3还可见于细胞间质脉管内皮细胞。每例切片至少计数10个400倍视野，阳性细胞数 < 1%记为0分，1%-25%为1分，26%-50%为2分，51%-75%为3分。按染色强度计分，淡棕褐色记1分，棕褐色记2分，深棕褐色记3分。以两者相加所得总分进行判定，得分≤2分记为表达阴性， > 2分记为表达阳性；由不相同的5位技术人员分别计算1次每组的表达阳性率，各组5次计算结果的平均值即为该组的表达阳性率。评估肿瘤组织中淋巴管生成的重要指标是组织中的微淋巴管密度，podoplanin蛋白是一种表达于脉管系统内皮细胞内的特异蛋白，对识别淋巴管内皮细胞具有较高的特异性，在NSCLC组织中，podoplanin蛋白是合适的淋巴管标记物，免疫组织化学染色能使表达有podoplanin蛋白的微淋巴管内皮细胞着色^[15, 16]^，再通过高倍镜下进行微淋巴管计数，五个不同视野的平均值作为微淋巴管密度。免疫磁珠负性富集法筛选出外周血循环肿瘤细胞，免疫荧光染色确定CTC，镜下观察CTC形态，计数各组CTC数量，比较各组差异。

### 1.4 统计学方法

采用SPSS 13.0统计软件，统计方法采用方差分析（*One*-*Way ANOVA*），其中包括*Levene*方差齐性检验，变量之间的相关性分析采用*Spearman*相关系数和*Kendall*相关系数评价，相关系数大于0.5提示为存在正相关，以*P* < 0.05为差异有统计学意义。

## 2 结果

### 2.1 肿瘤组织和淋巴结病理诊断

各组肿瘤组织和淋巴结制作成病理切片后进行HE染色，镜下观察肿瘤组织HE染色结果符合肺腺癌病理改变，淋巴结组织染色结果符合转移癌组织病理改变（图 1）。

**1:**
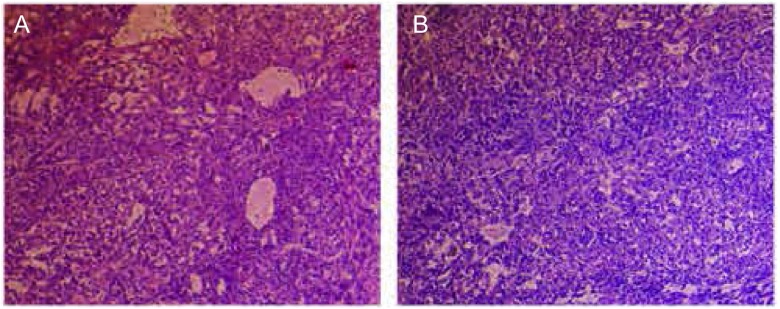
肿瘤组织和转移淋巴结HE染色图片（×100）。A：肿瘤组织HE染色图片；B：转移淋巴结HE染色图片。镜下所见肿瘤组织HE染色结果符合肺腺癌病理改变，淋巴结组织HE染色结果符合转移癌组织病理改变，病理诊断为肺腺癌和肺癌淋巴结转移。 HE staining picture of tumor tissue and metastatic lymph nodes (×100). A: HE staining picture of tumor tissue; B: HE staining picture of metastatic lymph nodes. Endoscopic findings HE staining result of tumor tissue pathological changes in accordance with the pathological changes of lung adenocarcinoma, HE staining results of lymph node tissue were in line with metastatic cancer tissue pathological changes, pathological diagnosis were lung adenocarcinoma and lung cancer lymph node metastasis.

### 2.2 VEGF-C、VEGF-D、VEGFR-3表达和微淋巴管密度

对肿瘤组织进行免疫组织化学染色诊断，染色成功后组织切片中有VEGF-C、VEGF-D、VEGFR-3表达的部位和微淋巴管内皮细胞呈棕黄色（图 2-图 5）。计算出各组肿瘤组织VEGF-C、VEGF-D、VEGFR-3表达阳性率和微淋巴管密度，详见表 1。重组人血管内皮抑素1、重组人血管内皮抑素2、重组人血管内皮抑素3及重组人血管内皮抑素1+顺铂、重组人血管内皮抑素2+顺铂、重组人血管内皮抑素3+顺铂各个组的VEGF-C、VEGF-D、VEGFR-3表达阳性率和微淋巴管密度均明显低于空白对照组与顺铂组（*P* < 0.05）；3个重组人血管内皮抑素组与3个重组人血管内皮抑素+顺铂组中，较高浓度重组人血管内皮抑素组的VEGF-C、VEGF-D、VEGFR-3表达阳性率和微淋巴管密度低于较低浓度组（*P* < 0.05）；3个重组人血管内皮抑素组与3个重组人血管内皮抑素+顺铂组中，相同重组人血管内皮抑素浓度的对应两组间VEGF-C、VEGF-D、VEGFR-3表达阳性率和微淋巴管密度无统计学差异（*P* > 0.05）。各组微淋巴管密度和VEGF-C、VEGF-D、VEGFR-3表达阳性率之间存在正相关，*Spearman*相关系数和*Kendall*相关系数均大于0.5。

**2:**
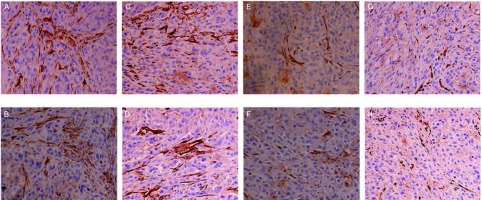
各组肿瘤组织经免疫组化处理微淋巴管着色后的图片（×400）。A：对照组；B：顺铂组；C：重组人血管内皮抑素1组；D：重组人血管内皮抑素2组；E：重组人血管内皮抑素3组；F：重组人血管内皮抑素1组+顺铂组；G：重组人血管内皮抑素2组+顺铂组；H：重组人血管内皮抑素3组+顺铂组。 Coloring microlymphatic vessel pictures of tumor tissue by IHC in each group (×400). A: Control group; B: Cisplatin group; C: Endostar 1; D: Endostar 2; E: Endostar 3; F: Endostar 1+Cisplatin; G: Endostar 2+Cisplatin; H: Endostar 3+Cisplatin. IHC: immunohistochmeistry.

**3:**
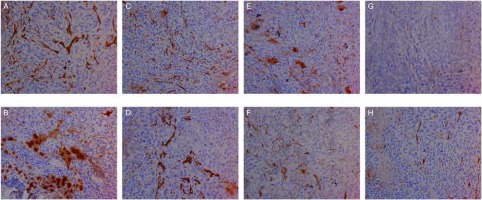
各组肿瘤组织经免疫组织化学染色VEGF-C着色后图片（×200）。A：对照组；B：顺铂组；C：重组人血管内皮抑素1组；D：重组人血管内皮抑素2组；E：重组人血管内皮抑素3组；F：重组人血管内皮抑素1组+顺铂组；G：重组人血管内皮抑素2组+顺铂组；H：重组人血管内皮抑素3组+顺铂组。 Coloring VEGF-C pictures of tumor tissue by IHC staining in each group (×200). A: Control group; B: Cisplatin group; C: Endostar 1; D: Endostar 2; E: Endostar 3; F: Endostar 1+Cisplatin; G: Endostar 2+Cisplatin; H: Endostar 3+Cisplatin. VEGF: vascular endothelial growth factor.

**4:**
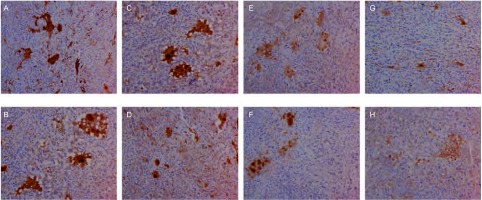
各组肿瘤组织经免疫组织化学染色VEGF-D着色后图片（×200）。A：对照组；B：顺铂组；C：重组人血管内皮抑素1组；D：重组人血管内皮抑素2组；E：重组人血管内皮抑素3组；F：重组人血管内皮抑素1组+顺铂组；G：重组人血管内皮抑素2组+顺铂组；H：重组人血管内皮抑素3组+顺铂组。 Coloring VEGF-D pictures of tumor tissue by IHC staining in each group (×200). A: Control group; B: Cisplatin group; C: Endostar 1; D: Endostar 2; E: Endostar 3; F: Endostar 1+Cisplatin; G: Endostar 2+Cisplatin; H: Endostar 3+Cisplatin.

**5:**
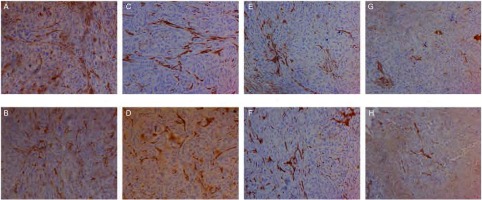
各组肿瘤组织经免疫组织化学染色VEGFR-3着色后图片（×200）。A：对照组；B：顺铂组；C：重组人血管内皮抑素1组；D：重组人血管内皮抑素2组；E：重组人血管内皮抑素3组；F：重组人血管内皮抑素1组+顺铂组；G：重组人血管内皮抑素2组+顺铂组；H：重组人血管内皮抑素3组+顺铂组。 Coloring VEGFR-3 pictures of tumor tissue by IHC staining in each group (×200). A: Control group; B: Cisplatin group; C: Endostar1; D: Endostar 2; E: Endostar 3; F: Endostar 1+Cisplatin; G: Endostar 2+Cisplatin; H: Endostar 3+Cisplatin.

**1:** 各组肿瘤组织中VEGF-C、VEGF-D、VEGFR-3表达阳性率，肿瘤组织微淋巴管密度以及循环肿瘤细胞数目 Positive expression rate of VEGF-C, VEGF-D, VEGFR-3, microlymphatic vessel density of tumor tissues and CTC number in each group

Group	Sample size	Positive rate			MLVD	CTC number
		VEGF-C	VEGF-D	VEGFR-3		
Control group	7	94.3%	91.4%	88.6%	9.94±0.19	25.43±1.98
Cisplatin group	7	91.4%	88.6%	85.7%	9.29±0.29	7.14±1.95
Endostar 1	7	65.7%	62.9%	68.6%	6.30±0.34	15.13±2.17
Endostar 2	7	48.6%	42.6%	51.4%	5.55±0.39	10.63±1.60
Endostar 3	7	31.4%	28.6%	34.3%	4.33±0.29	6.25±1.67
Endostar 1+Cisplatin	7	62.9%	57.1%	65.7%	6.39±0.29	3.13±0.83
Endostar 2+Cisplatin	7	45.7%	40.0%	48.6%	5.48±0.31	1.00±0.76
Endostar 3+Cisplatin	7	28.6%	25.7%	31.4%	4.35±0.25	0.13±0.35
VEGF: vascular endothelial growth factor; VEGFR: VEGF receptor; MLVD: microlymphatic vessel density; CTC: circulating tumor cell.						

### 2.3 外周血CTC检测

免疫磁珠负性富集法筛选出外周血循环肿瘤细胞，采用免疫荧光染色，共聚焦显微镜下观察CTC形态（图 6），计数CTC数目，8组中的7个组外周血中检测出了CTC，详见表 1。重组人血管内皮抑素1组、重组人血管内皮抑素2组、重组人血管内皮抑素3组及重组人血管内皮抑素1+顺铂、重组人血管内皮抑素2+顺铂、重组人血管内皮抑素3+顺铂和顺铂组的CTC数目明显低于空白对照组（*P* < 0.01），3个重组人血管内皮抑素组与3个重组人血管内皮抑素+顺铂组中，较高浓度重组人血管内皮抑素组的CTC数目低于较低浓度组（*P* < 0.01），重组人血管内皮抑素联合顺铂各组的CTC数目明显低于单独使用顺铂或重组人血管内皮抑素（*P* < 0.01）。

**6:**
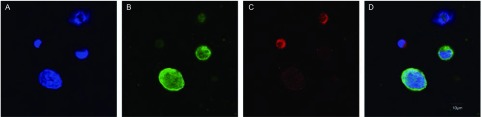
共聚焦显微镜下循环肿瘤细胞图片，图中标尺代表 10 μm，×1, 000。A：循环肿瘤细胞经细胞核荧光染料DAPI染色的图片；B：循环肿瘤细胞经anti-NapsinA荧光抗体染色的图片；C：循环肿瘤细胞经anti-TTF-1荧光抗体染色的图片；D：图 6A、B、C整合而成的图片。 Confocal microscopy images of circulating tumor cells, the scale bar represents 10 μm, ×1, 000. A was an image of circulating tumor cells dyed by a nucleus fluorescent dye called DAPI; B was an image of circulating tumor cells dyed by anti-NapsinA fluorescent antibody; C was an image of circulating tumor cells dyed by anti-TTF-1 fluorescent antibody; D was an image of circulating tumor cells integrated from Fig 6A-C.

## 3 讨论

肺癌是发病率和死亡率增长最快，对人群健康和生命威胁最大的恶性肿瘤之一，多数肺癌患者死于转移，淋巴转移是肿瘤转移的主要途径之一，部分肿瘤患者在发现原发病灶的同时，已经出现了多处的淋巴结转移^[17]^。早期的观点普遍认为肿瘤通过周围已有的淋巴管转移，随着研究的深入发现，肿瘤可以诱导淋巴管生成从而促进转移^[18, 19]^。研究^[20-22]^显示，VEGF-C、VEGF-D和VEGFR-3与肿瘤淋巴结生成和转移的关系极为密切，VEGF-C、VEGF-D是淋巴管生成有力的诱导剂，可通过结合并激活特异性表达于淋巴管内皮的VEGFR-3，介导和启动受体的酪氨酸激酶VEGF-C、VEGF-D/VEGFR-3信号通道，诱导内皮细胞增殖、迁移和特异性淋巴内皮细胞生长，促进肿瘤淋巴管的生成和淋巴管扩张，增加入侵癌细胞与淋巴管的接触面积，促进肿瘤淋巴转移。迄今为止，有关血管内皮抑素对肿瘤淋巴管生成抑制作用的动物实验及临床研究并不多见。肿瘤的淋巴转移途径与血行转移同等重要，因此研究阻断肿瘤淋巴转移途径的方法同样意义重大。

本实验创新点即在于研究重组人血管内皮抑素对肺癌动物模型新生淋巴管的抑制作用，可以为血管生成抑制因子如重组人血管内皮抑素能否抑制肿瘤淋巴转移提供实验依据。综合实验中各组VEGF-C、VEGF-D、VEGFR-3表达阳性率和微淋巴管密度检测结果的差异，以及微淋巴管密度与VEGF-C、VEGF-D、VEGFR-3表达阳性率的相关性，可以认为重组人血管内皮抑素确实具有抑制肿瘤淋巴管生成的作用，通过抑制肿瘤组织中VEGF-C、VEGF-D的表达，减少组织中VEGFR-3的激活，抑制VEGF-C、VEGF-D/VEGFR-3信号通道，达到抑制肿瘤淋巴管生成和淋巴转移的目的。

Sleeman等^[1]^认为，肿瘤发生早期进入血管的少量肿瘤细胞会因为血液中的血流动力学压力等因素而大部分被破坏，而淋巴管中淋巴液流动相对缓慢，侵入淋巴道的肿瘤细胞则可以继续增殖，先造成淋巴结转移，并在转移淋巴结内分化增殖，最终大量的肿瘤细胞通过胸导管进入血液循环达到全身组织器官而形成远处转移。实验中各组外周血循环肿瘤细胞检测结果，提示重组人血管内皮抑素与顺铂均能减少循环肿瘤细胞数目，但作用机制不同，重组人血管内皮抑素通过抑制肿瘤血管与淋巴管生成，减少肿瘤细胞进入血液循环，作用大小与给药浓度呈正相关；顺铂则是通过杀死进入血液循环的肿瘤细胞，从而减少循环肿瘤细胞。重组人血管内皮抑素与顺铂联合使用相比单独使用重组人血管内皮抑素或顺铂能更有效的减少循环肿瘤细胞数目。采用重组人血管内皮抑素联合顺铂，能够更好地抑制肿瘤通过血液和淋巴道转移，降低肿瘤复发和转移的风险。

综上所述，重组人血管内皮抑素抑制NSCLC裸鼠模型淋巴生成的基础研究，证实了重组人血管内皮抑素可通过抑制肺癌组织中VEGF-C、VEGF-D、VEGFR-3的表达来抑制新生淋巴管形成，抑制肺癌的淋巴转移；同时可以减少肿瘤细胞经淋巴管进入血液循环，与顺铂联合使用可更有效降低肺癌远处转移和复发的风险。重组人血管内皮抑素抑制NSCLC裸鼠模型淋巴生成的基础研究，不仅对NSCLC患者的治疗具有指导作用，也为易发生淋巴转移的其他恶性肿瘤提供了新的用药选择。希望通过进一步的基础与临床研究，让更多的恶性肿瘤患者获益。
